# Peripheral chemoreflex modulation of renal hemodynamics and renal tissue PO2 in chronic heart failure with reduced ejection fraction

**DOI:** 10.3389/fphys.2022.955538

**Published:** 2022-08-26

**Authors:** Kiefer W. Kious, Andrew Philipose, Luke J. Smith, Jayson P. Kemble, Stephanie C. E. Twohey, Kalie Savage, Hugo S. Díaz, Rodrigo Del Rio, Noah J. Marcus

**Affiliations:** ^1^ Department of Physiology and Pharmacology, Des Moines University Medicine and Health Sciences, Des Moines, IA, United States; ^2^ Department of Biology, Simpson College, Indianola, IA, United States; ^3^ Laboratory of Cardiorespiratory Control, Department of Physiology, Pontificia Universidad Católica de Chile, Santiago, Chile

**Keywords:** heart failure, cardio renal syndrome (CRS), carotid body chemoreflex, carotid body denervation (CBD), renal blood flow (RBF), renal oxygenation

## Abstract

Aberrant carotid body chemoreceptor (CBC) function contributes to increased sympathetic nerve activity (SNA) and reduced renal blood flow (RBF) in chronic heart failure (CHF). Intermittent asphyxia (IA) mimicking sleep apnea is associated with additional increases in SNA and may worsen reductions in RBF and renal PO2 (RPO2) in CHF. The combined effects of decreased RBF and RPO2 may contribute to biochemical changes precipitating renal injury. This study sought to determine the role of CBC activity on glomerular filtration rate (GFR), RBF and RPO2 in CHF, and to assess the additive effects of IA. Furthermore, we sought to identify changes in gene expression that might contribute to renal injury. We hypothesized that GFR, RBF, and RPO2 would be reduced in CHF, that decreases in RBF and RPO2 would be worsened by IA, and that these changes would be ameliorated by CBC ablation (CBD). Finally, we hypothesized that CHF would be associated with pro-oxidative pro-fibrotic changes in renal gene expression that would be ameliorated by CBD. CHF was induced in adult male Sprague Dawley rats using coronary artery ligation (CAL). Carotid body denervation was performed by cryogenic ablation. GFR was assessed in conscious animals at the beginning and end of the experimental period. At 8-weeks post-CAL, cardiac function was assessed via echocardiography, and GFR, baseline and IA RBF and RPO2 were measured. Renal gene expression was measured using qRT-PCR. GFR was lower in CHF compared to sham (*p* < 0.05) but CBD had no salutary effect. RBF and RPO2 were decreased in CHF compared to sham (*p* < 0.05), and this effect was attenuated by CBD (*p* < 0.05). RBF and RPO2 were reduced to a greater extent in CHF vs. sham during exposure to IA (*p* < 0.05), and this effect was attenuated by CBD for RBF (*p* < 0.05). Downregulation of antioxidant defense and fibrosis-suppressing genes was observed in CHF vs. sham however CBD had no salutary effect. These results suggest that aberrant CBC function in CHF has a clear modulatory effect on RBF during normoxia and during IA simulating central sleep apnea.

## Introduction

Thirty to forty percent of patients with chronic heart failure (CHF) develop co-morbid renal dysfunction (Zannad and Rossignol, 2018; Costanzo, 2022). This “Cardio-Renal syndrome” (CRS) is characterized by a positive feedback loop between the heart and kidneys in which dysfunction in one organ precipitates or exacerbates dysfunction in the other (Zannad and Rossignol, 2018; Costanzo, 2022). CRS has become an area of intense interest due to the complexity of therapeutic management and associated morbidity and mortality (Zannad and Rossignol, 2018; Costanzo, 2022). Current theories of CRS type II etiology in chronic heart failure (CHF) suggest that low cardiac output, sustained increases in sympathetic nerve activity, and activation of the renin-angiotensin-aldosterone system (RAAS) combine to produce venous congestion and increased abdominal pressure, and that all of these together contribute to renal hypoperfusion, hypoxia, and tissue injury (Zannad and Rossignol, 2018; Costanzo, 2022; Patel et al., 2022). Recent theories suggest that tissue injury leading to development of organ fibrosis is a common unifying factor in development of all types of CRS (Zannad and Rossignol, 2018).

Autonomic dysfunction is a hallmark of CHF and plays a central role in CRS pathophysiology as tonic increases in sympathetic nerve activity reduce renal blood flow and venous capacitance, and cause RAAS activation and related downstream pro-oxidative and pro-fibrotic biochemical signaling pathways (Zannad and Rossignol, 2018; Costanzo, 2022; Patel et al., 2022). Abundant evidence from human and animal studies shows that abnormal autonomic function in CHF results from altered function in peripheral cardiovascular reflexes (baroreflex, chemoreflex, cardiac afferent reflex, muscle mechanoreflex) as well as central neural pathways that regulate autonomic function (Ponikowski et al., 1997; Giannoni et al., 2008; Del Rio et al., 2013; Marcus et al., 2014; Wang et al., 2014; Xing et al., 2014; Tubek et al., 2021; Patel et al., 2022). Earlier work from our lab has shown that chemoreflex-mediated increases in renal sympathetic nerve activity (RSNA) contribute to reductions in resting RBF and exaggerated RBF responses to hypoxia in rabbits with pacing-induced CHF (Marcus et al., 2014; Marcus et al., 2015; Pügge et al., 2016).

Chemoreflex-mediated increases in RSNA contribute to decreased oxygen delivery by constraining renal blood flow and increasing renal venous pressure. In addition, increased RSNA and decreased RBF may lead to increased renal oxygen consumption through RAAS activation and increased sodium avidity (Johns et al., 2011), The combination of these stimuli results in reduced renal tissue PO2. Renal PO2 (RPO2) has been extensively studied as a potential contributor to renal injury in the context of chronic kidney disease and hypertension (Honda et al., 2019), however its role in CRS is not well defined. To date no studies have attempted to measure RPO2 in CHF or its association with changes in renal hemodynamics. Therefore, the primary purpose of this study was to determine if RPO2 is reduced in CHF and to determine if modulation of CB chemoreceptor (CBC) activity has salutary effects. Because CHF patients often have co-morbid sleep apnea, and the intermittent asphyxia (IA) associated with sleep apnea is associated with enhanced afferent and efferent chemoreflex activity (Naughton et al., 1995; Peng et al., 2003; Marcus et al., 2010; Del Rio et al., 2014), a second aim of this study was to determine if RBF and RPO2 responses to intermittent asphyxia are altered in CHF. Finally, tissue injury and decline in renal function typically result from a combination of insults in type II CRS (Zannad and Rossignol, 2018; Costanzo, 2022), therefore we also sought to identify changes in renal cortical gene expression related to sympathetic activation, declines in blood flow, and tissue hypoxia that might contribute to oxidative stress and development of fibrosis.

We hypothesized that RBF and RPO2 would be concomitantly reduced in CHF and that the compound effects of IA and CHF on CB chemoreflex function would worsen decreases in RBF and RPO2 during IA. We also hypothesized that antioxidant defense genes and fibrosis-suppressing gene programs would be downregulated in CHF and attenuated by CBD. To address these aims we measured RBF and RPO2 in CHF rats with and without ablation of the CBC at rest and during exposure to ten episodes of IA. We measured gene expression of transcription factors and their downstream targets related to antioxidant defense as well as genes related to tissue fibrosis. Our results show a link between CHF and chemoreflex function that facilitates development of CRS through chronic reductions in RBF and RPO2 which is exacerbated by IA. We also noted changes in renal cortical gene expression in CHF that contribute to decline in renal function.

## Materials and methods

### Ethical approval & statement of compliance

The experimental protocols were approved by the Des Moines University Institutional Animal Care and Use Committee and were carried out in accordance with the National Institutes of Health (NIH Publication No. 85-23, revised 1996) and the American Physiological Society’s Guide for the Care and Use of Laboratory Animals. The authors wish to declare that our work complies with the animal ethics checklist outlined by Grundy (Grundy, 2015).

### Experimental groups

**FIGURE 1:**
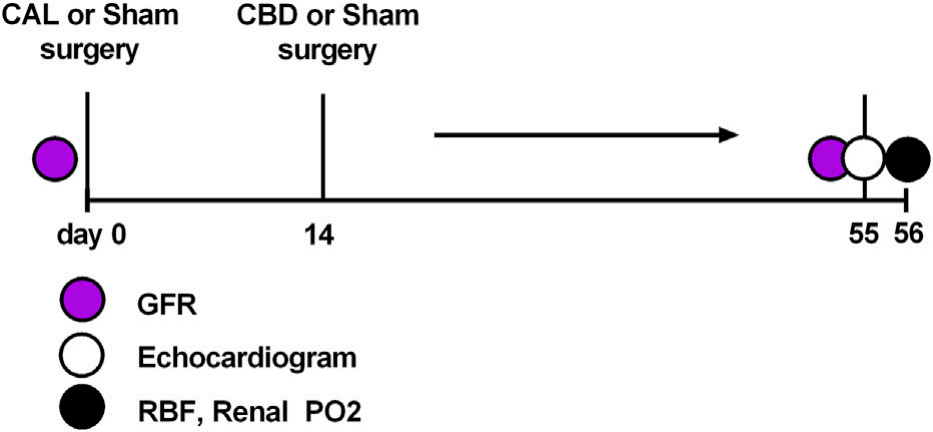
Experimental timeline.

Male Sprague-Dawley rats (Envigo, Madison, WI) of similar age (8–10 weeks) and weight (250–300 g) were used for these experiments. All rats were group-housed under controlled temperature (23°C) and humidity conditions and kept on a 12:12 h light-dark cycle and fed standard rat chow with water available *ad libitum*. At the beginning of the study animals were randomly assigned to one of three groups: sham (animals that underwent thoracotomy with sham ligation and sham denervation surgery), CHF (animals that underwent thoracotomy with coronary artery ligation and sham denervation surgery), CHF-CBD (animals that underwent thoracotomy with coronary artery ligation and carotid body denervation). At the onset of the study 53 animals were randomly assigned to the aforementioned groups (20 in each CHF group, and 13 in the sham CHF group). The number of animals assigned to each group was based on power analysis with allowance for unsuccessful ligation, perioperative mortality, or unanticipated technical difficulties. The number of animals/observations in each experiment is indicated in the respective figure legends. All groups followed similar timelines as shown in Figure 1.

### Anesthetic protocol & monitoring procedures

For all surgeries, anesthesia was induced with 5% inhalation isoflurane in air after which time anesthesia was reduced to 1.5%–3% as necessary to maintain a surgical plane of anesthesia. In order to determine appropriate anesthetic depth was reached, a toe pinch and/or tail pinch was administered after induction and every 15 min thereafter. The absence of paw withdrawal, movement of the tail, vocalization, or marked increase in respirations after a pinch was taken as evidence of adequate anesthetic depth. Body temperature was measured using a rectal thermometer and monitored using a Physiosuite w/RightTemp Temperature Monitoring and Homeothermic Control system (Kent Scientific, CT), and was maintained at 37°C using a far infrared heating pad integrated with a Surgisuite surgical platform (Kent Scientific, CT).

### Surgical procedures for induction of chronic heart failure

Heart failure was induced via coronary artery ligation (CAL) as described previously (Del Rio et al., 2013; Wang et al., 2014). A brief description of the procedure follows. Rats were anesthetized with isoflurane (1.5% isoflurane with 21% O2) and then underwent endotracheal intubation (Kent Scientific Co., Torrington, CT, Intubation Kit) and mechanical ventilation (SAR-1000 Ventilator, Ardmore, PA). Tidal volume and ventilation rate were determined according to animal weight (SAR-1000 manual guidelines), and anesthetic dose was controlled using the SomnoSuite (Kent Scientific Co.) anesthesia system. After rats were successfully anesthetized, intubated, and ventilated, an incision was made in the fifth intercostal space and the heart exposed. The pericardium was removed, and a 6-0 Prolene suture was used to tie a ligature around the left anterior descending coronary artery. The thorax was closed in layers using 3-0 Polysorb for the subcutaneous closure and surgical staples for closure of the skin. A chest tube was inserted to evacuate the chest following closure. Animals were maintained on a heating pad until they regained sternal recumbency. Sham animals underwent the same procedure except coronary artery ligation was not performed. Post-operative analgesia was provided via subcutaneous injection of 0.05 mg/kg Buprenorphine and 4 mg/kg Carprofen. Doses of Carprofen were repeated every 24 h post-surgery (minimum of 48 h post-surgery, but longer as needed).

### Carotid body denervation

Carotid body denervation (CBD) was performed under isoflurane anesthesia 2-weeks post-CAL as described previously (Del Rio et al., 2013). Briefly, following a ventral incision on the surface of the neck and isolation of the bifurcation of the carotid artery, the carotid body was identified. In the CHF-CBD group, the CB on both sides was cryogenically destroyed using a fine tipped metal probe cooled by liquid nitrogen. Sham and CHF groups underwent the same surgical procedures without cryogenic ablation of the carotid bodies. Post-operative analgesia was provided via subcutaneous injection of 4 mg/kg Carprofen. Doses of Carprofen were repeated every 24 h post-surgery (minimum of 48 h post-surgery, but longer as needed). The criteria for successful CBD in this study was an attenuation of the increase in the ventilatory response to hypoxia. Based on our previous studies in this experimental model (Del Rio et al., 2013) we set this threshold at a ∆Ve of less than or equal to 30 ml/min/100 g bw.

### Measurement of glomerular filtration rate

GFR was assessed non-invasively in conscious, unrestrained rats by measuring clearance of the fluorescent tracer FITC-sinistrin as described previously (Schock-Kusch et al., 2009; Schock-Kusch et al., 2011; Friedemann et al., 2016; Herrera Pérez et al., 2016). FITC-sinistrin clearance was measured transdermally using the NIC-kidney device (Medibeacon, St. Louis MO) affixed to the rat’s back. On the day prior to the study, a site on the back was shaved and prepared with a depilatory agent (i.e., Nair). On the day of the study the rats were anesthetized (1.5% Isoflurane) and the depilated area was cleaned. The monitor was affixed to the back with an adhesive patch and a bolus injection of FITC-sinistrin (40 mg/ml) was given through one of the lateral tail veins. Measurements of FITC-sinistrin fluorescence were made once per second for 120 min. At the conclusion of the experiment, the monitor and battery pack were removed, and the data was downloaded for later analysis with MPD Lab software (Medibeacon, St Louis MO) as described previously (Friedemann et al., 2016). Measurements were taken prior to thoracotomy and at the conclusion of the study (8-weeks post-thoracotomy).

### Assessment of cardiac function

Cardiac function was quantified 8 weeks after CAL (or sham CAL) surgery using echocardiography (Mindray Diagnostic Ultrasound System, Duluth, GA, model: M6, with C11-3s ultrasonic transducer) under isoflurane anesthesia (1.5%). Three to four consecutive measurements of left ventricle end-systolic diameter (LVESD) and left ventricle end-diastolic diameter (LVEDD) were averaged, in accordance with the guidelines set forth by the American Society of Echocardiography (Lang et al., 2015). Using the Teicholz method ([LVESV = 7*LVESD3/(2.4 + LVESD] and [LVEDV = 7*LVEDD3/(2.4 + LVEDD], left ventricular end-systolic volume (LVESV) and left ventricular end-diastolic volume (LVEDV) were calculated (Lang et al., 2015). Ejection fraction and fractional shortening (FS) were then calculated from the acquired left ventricular diameters (Lang et al., 2015). All measurements were obtained using parasternal long axis view.

### Measurement of Renal Blood Flow and Renal PO2

Rats were anesthetized with isoflurane (1.5%–2% in air). The body temperature of the animal was maintained with a far infrared heating pad during experiments (Surgisuite, Kent Scientific, Torrington, CT). The left femoral artery was cannulated with a PE10 catheter for measurement of arterial pressure with a differential pressure transducer and bridge amp (AD Instruments). Then a ventral mid-line incision was made, and the kidney was approached in the peritoneal space. Once the renal artery and vein were identified the renal artery was gently separated from the vein and a transit-time flow probe (Transonic, Ithaca, NY) was placed around the renal artery. After the flow probe was secured, a needle probe was inserted 1–2 mm into the kidney to measure cortical RPO2 using the Oxylite Pro (Oxford Optronix, United Kingdom). After a 60-min equilibration period a 5-min baseline period of RBF and RPO2 was recorded for analysis.

After measurement of baseline values, a series of ten 30 s episodes of intermittent asphyxia (IA) were initiated. Each IA episode was characterized by an FiO_2_ of 10% and FiCO_2_ 3% with the remaining balance Nitrogen. Each IA episode was followed by a 60 s recovery period breathing medical grade air (FiO_2_ 21%, balance Nitrogen). All variables were measured continuously during the IA protocol.

### Method of euthanasia

At the conclusion of the experimental protocol all rats were humanely euthanized. In accordance with standards set forth by the American Veterinary Medical Association and the Institutional Animal Care and Use Committee at Des Moines University, all rats were euthanized via anesthetic overdose (5% isoflurane) with the addition of a secondary physical method of euthanasia (bilateral thoracotomy).

### Measurement of renal cortical mRNA expression

Rat kidneys were immediately removed after euthanasia and cortical samples were dissected and frozen in liquid nitrogen and stored at −80°C for later analysis. RNA isolation and cDNA synthesis were performed according to manufacturer instructions using the Direct-zol RNA MiniPrep Plus (Zymo) and High-Capacity cDNA Reverse Transcriptase (Thermo Fisher Scientific) kits, respectively. Gene expression of Kruppel-like factor 2 (KLF2), endothelial nitric oxide synthase (eNOS), Nuclear factor erythroid 2-related factor 2 (NRF2), NAD(P)H quinone dehydrogenase 1 (NQO1), Kruppel-like Factor 15 (KLF15), E Cadherin, Kruppel-like Factor 4 (KLF4), and connective tissue growth factor (CTGF) was assessed by Sso Advanced SYBR green chemistry (Bio-Rad) real-time PCR following reverse transcription of total RNA. Real-time PCR was performed on a CFX Connect Real Time PCR Detection System (Bio-Rad, Hercules, CA). β-Actin mRNA was quantified as an internal control for each sample and quantifications were performed using the ΔΔCt method and expressed as fold change (f.c.). Forward and Reverse primer sequences are show in Table 2.

### Data analysis

All physiological parameters other than GFR and echocardiography were recorded with a Powerlab data acquisition and analysis system. Mean values for baseline RBF and RPO2 were calculated from 5-min of data immediately following a 60-min post-instrumentation equilibration period. To quantify the RBF and RPO2 response to IA we used two approaches. First, we analyzed the nadir RBF and RPO2 during IA. Second, we analyzed the average change (delta) in RBF and RPO2 (baseline to nadir) during the IA protocol. Taken together these results should give a complete picture of the absolute levels of RBF and RPO2 (at their lowest point) as well as the magnitude of change in each group over the course of the IA protocol. All data was tested for normality using D’Agostino & Pearson omnibus normality test and the Shapiro-Wilk normality test. If normality tests were passed, parametric statistics were used, otherwise non-parametric analyses were used. Cross-sectional group mean data was analyzed using one-way analysis of variance or Kruskal-Wallis (for non-parametric data). Holm-Šídák or Dunn’s tests were used for pairwise comparisons for ANOVA and Kruskal Wallis, respectively. For determination of successful induction of CHF, we used a minimum cutoff of 40% below the ejection fraction observed in sham animals, and animals in CHF groups not meeting this criteria were excluded from all analyses. All data was analyzed using GraphPad Prism software (San Diego CA). All data are expressed as mean ± standard error of the mean (SEM). Statistical significance was accepted when *p* < 0.05.

## Results

### Effect of carotid body denervation on cardiac and respiratory function in chronic heart failure

Echocardiographic measurements (shown in Table 1) were taken at the end of the experimental period prior to experiments to measure RBF and RPO2. Ejection fraction (EF), fractional shortening (FS), and stroke volume (SV) were significantly lower (*p* < 0.05) in CHF and CHF-CBD groups relative to sham, however there were no significant differences between CHF and CHF-CBD groups. Body weight (BW) prior to physiological experiments (Table 1), was not significantly different between sham, CHF, and CHF-CBD groups. In order to determine the efficacy of CBD, the ventilatory response to isocapnic hypoxia (2 min at FiO2 10%, FiCO2 3%) was measured in conscious animals using barometric plethysmography as previously described (Del Rio et al., 2013). In sham animals the mean increase in minute ventilation from baseline was 40 ± 5 ml/min/100 g bw, the mean increase in minute ventilation in CHF-sham animals was 60 ± 5 ml/min/100 g bw, and the mean increase in minute ventilation in CHF-CBD animals was 20 ± 4 ml/min/100 g bw (*p* < 0.05 between all groups, via one way ANOVA and Sidak-Holm multiple comparison test, F 37.06 DF 2,27).

**TABLE 1:** Echocardiographic measurements and baseline cardiovascular variables.

sham	sham	CHF	CHF-CBD	(F, DF)
Body Weight (kg)	408.4 ± 10.3	387.6 ± 12.1	413.2 ± 15.1	(1.48, 2,27)
LVd Vol (ml)	0.68 ± 0.07	0.50 ± 0.07	0.42 ± 0.06	(6.77, 2,27)
LVs Vol (ml)	0.11 ± 0.02	0.26 ± 0.05*	0.20 ± 0.04	(8.23, 2,27)
SV (ml)	0.57 ± 0.07	0.23 ± 0.03*	0.22 ± 0.04*	(51.68, 2,27)
EF (%)	83.5% ± 2.36%	47.7% ± 2.9%*	49.5% ± 2.4%*	(91.54, 2,27)
FS (%)	51.4% ± 1.4%	22.6% ± 1.6%*	23.2% ± 1.4%*	(125.4, 2,27)
MAP (mmHg)	76 ± 8	83 ± 5	78 ± 7	(0.50, 2,27)
HR (beats/min)	310 ± 18	313 ± 15	300 ± 12	(0.24, 2,27)

LVd Vol, left ventricular diastolic volume; LVs Vol, left ventricular systolic volume; SV, stroke volume; EF%, ejection fraction; FS%, fractional shortening; MAP, mean arterial pressure; HR, heart rate. Data was analyzed using one-way ANOVA, with Sidak-Holm multiple comparison test. Results are expressed as mean ± SEM, *n* = 10 animals per group. *p < 0.05 vs. sham.

### Effect of carotid body denervation on glomerular filtration rate in chronic heart failure

**FIGURE 2:**
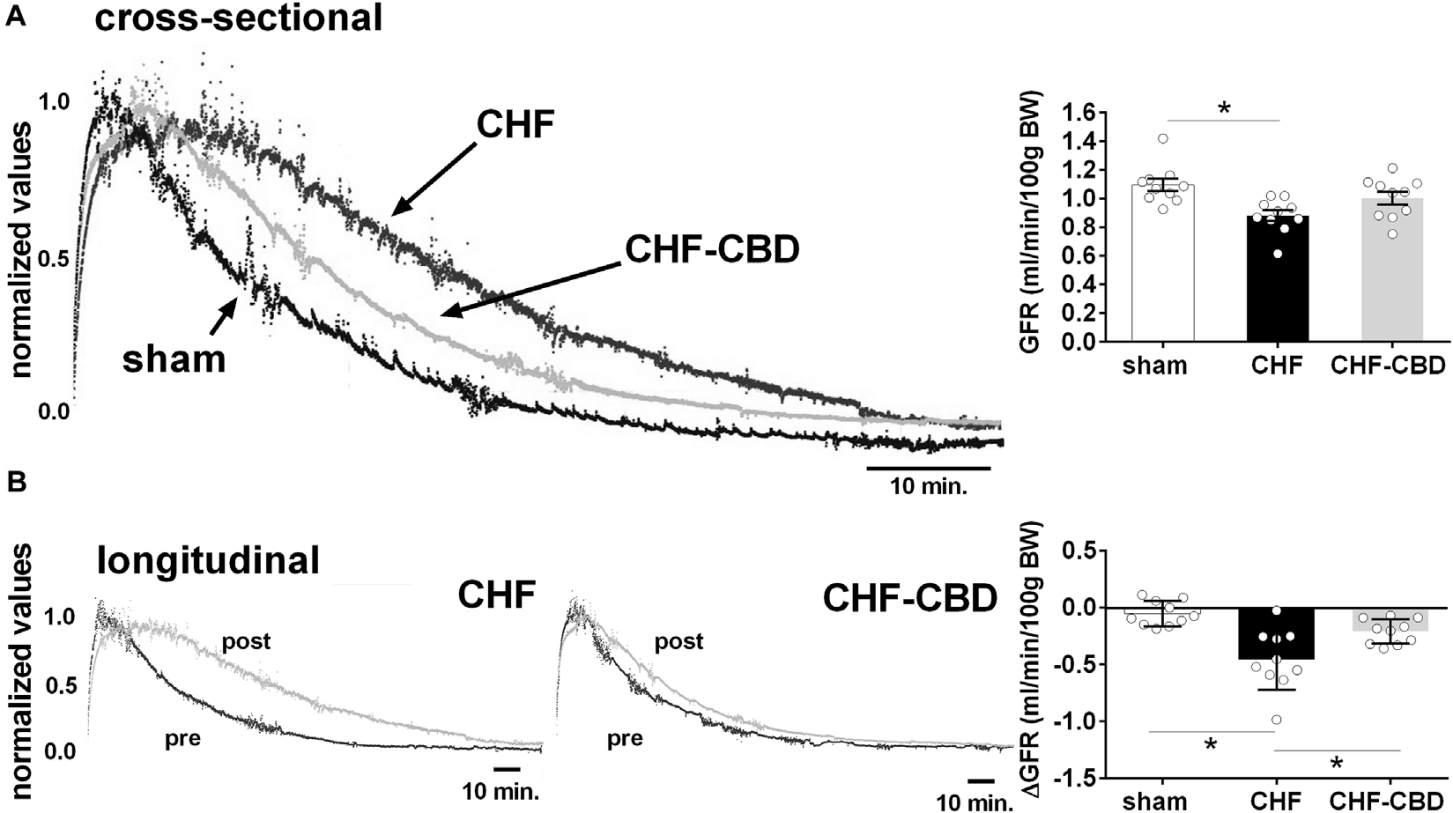
Effect of CHF and CBD on Glomerular Filtration Rate. Shown in panel **(A)** are representative tracings of FITC fluorescence decay curves in one sham, CHF, and CHF-CBD rat. Shown in the right panel are summary data illustrating the effect of CHF and CHF with CBD on GFR. Note GFR was significantly lower in CHF vs. sham, but that GFR in CHF-CBD was not significantly higher than in CHF. Panel **(B)** represents longitudinal assessment (pre and post) of GFR in one CHF and one CHF-CBD rat. Shown in the right panel are summary data illustrating the analysis of change in GFR from pre to post condition in each experimental group. Results are expressed as mean ± SEM. *n* = 10 animals per group. *p < 0.05 vs. sham via ANOVA with Sidak-Holm multiple comparison tests.

Glomerular filtration rate (GFR) when compared cross-sectionally across experimental groups was significantly lower in the CHF group than sham (*p* < 0.05), whereas GFR in the CHF-CBD was not significantly different from either sham or CHF (1.09 ± 0.04 sham, 0.88 ± 0.04 CHF, 1.00 ± 0.04 CHF-CBD, one way ANOVA and Sidak Holm multiple comparison test, F 6.47 DF 2,27, Figure 2A). For longitudinal comparisons, the delta GFR was calculated between pre-post measurements for each animal and group means were compared. The decrease in GFR from pre to post was significantly greater (*p* < 0.05) in CHF vs. sham, and significantly less (*p* < 0.05) in CHF-CBD vs. CHF (−0.05 ± 0.03 sham, −0.45 ± 0.08, CHF, −00.21 ± 0.03 CHF-CBD, one way ANOVA and Sidak Holm multiple comparison test, F 12.91 DF 2,27, Figure 2B).

### Effect of carotid body denervation on baseline renal blood flow and renal PO2 in chronic heart failure

**FIGURE 3:**
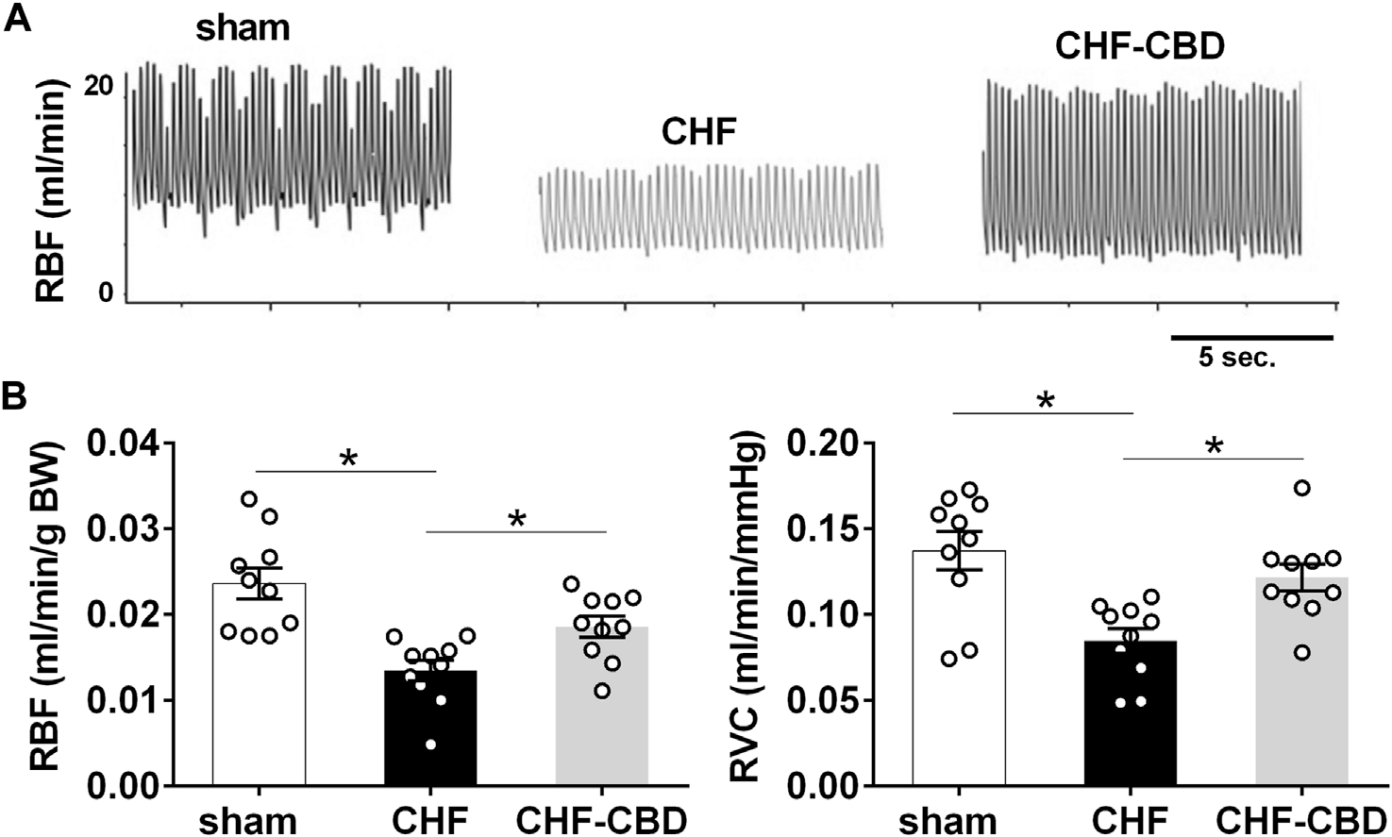
Reductions in Normoxic Renal Blood Flow in CHF are Attenuated by CBD. Shown in panel **(A)** are representative tracings of renal blood flow in one sham, one CHF, and one CHF-CBD rat. Shown in panel **(B)** are summary data illustrating the effect of CBD on RBF and conductance (RVC) in CHF. Note significant reductions in RBF&RVC in CHF that are attenuated by CBD. Results are expressed as mean ± SEM. *n* = 10 animals per group. *p < 0.05 vs. sham via ANOVA with Sidak-Holm multiple comparison tests.

**FIGURE 4:**
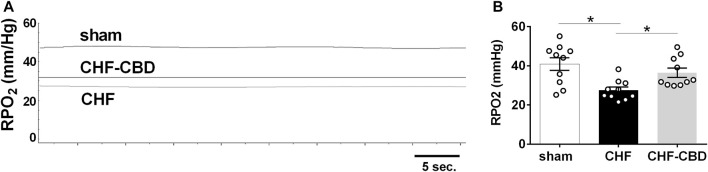
Reductions in Normoxic Renal PO2 (RPO2) in CHF are Attenuated by CBD. Shown in panel **(A)** are representative tracings of RPO2 in one sham, CHF, and CHF-CBD rat. Shown in panel **(B)** are summary data illustrating the effect of CBD on RPO2 in CHF. Note cortical RPO2 was significantly lower in CHF vs. sham, and that CBD resulted in higher RPO2 in CHF group. Results are expressed as mean ± SEM. *n* = 10 animals per group. *p < 0.05 vs. sham via ANOVA with Sidak-Holm multiple comparison tests.

RBF in sham, CHF, and CHF-CBD rats was measured at the conclusion of the experimental period. As show in Figure 3, RBF and RVC was significantly lower in CHF rats in relation to sham rats (*p* < 0.05), and this effect was attenuated in CHF-CBD rats as RBF and RVC was significantly higher (*p* < 0.05) in this group relative to CHF rats (RBF 0.024 ± 0.002 ml/min/gBW sham, 0.014 ± 0.001 ml/min/gBW CHF, 0.019 ± 0.001 ml/min/gBW CHF-CBD, F 12.24 DF 2,27; RVC 0.137 ± 0.002 ml/min/mmHg sham, 0.085 ± 0.007 ml/min/mmHg CHF, 0.122 ± 0.007 ml/min/mmHg, F 9.185 DF 2,27). Cortical RPO2 was measured concurrently with RBF measurements at the conclusion of the experimental period. As show in Figure 4 baseline cortical RPO2 was significantly lower in CHF relative to sham (*p* < 0.05), and this effect was attenuated in CHF-CBD as cortical RPO2 in this group was significantly higher (*p* < 0.05) than in CHF (RPO2 41 ± 3 mmHg sham, 28 ± 2 mmHg CHF, 37 ± 2 mmHg CHF-CBD, F 7.522 DF 2,27). Mean arterial pressure was quantified from the 5-min data segment used to assess baseline RBF and RPO2 (post-equilibration period). As shown in Table 2, no significant differences were observed between groups for baseline mean arterial pressure (MAP) or heart rate (HR).

**TABLE 2:** Primer sequences.

Primer	Forward	Reverse
β-Actin	GGA​GAT​TAC​TGC​CCT​GGC​TCC​TA	GAC​TCA​TCG​TAC​TCC​TGC​TTG​CTG
KLF2	ACT​TGC​AGC​TAC​ACC​AAC​TG	CTGTGACCCGTGTGCTTG
eNOS	TAT​TTG​ATG​CTC​GGG​ACT​GC	AAG​ATT​GCC​TCG​GTT​TGT​TG
NRF2	AAG​GTT​TCC​CAT​CTC​CAT​CAC	GAA​TAA​AGT​TGC​CGC​TCA​GAA
NQO1	CTC​GCC​TCA​TGC​GTT​TTT​G	CCC​CTA​ATC​TGA​CCT​CGT​TCA​T
KLF15	CAG​CTT​CTG​GTC​AAC​ATC​CA	GAA​GTT​CTG​CTG​CTG​GGT​TC
E Cadherin	AAA​GCA​GGA​AGA​AAA​CAC​CAC​TC	AAA​GGG​CAC​GCT​ATC​AAC​ATT​AG
KLF4	GAG​AGG​AAC​TCT​CTC​ACA​TGA​AGC	AAG​GAT​AAA​GTC​TAG​GTC​CAG​GAG​A
CTGF	CCT​GGT​CCA​GAC​CAC​AGA​GT	TTT​TCC​TCC​AGG​TCA​GCT​TC

### Effect of carotid body denervation on renal blood flow and renal PO2 during intermittent asphyxia in chronic heart failure

**FIGURE 5:**
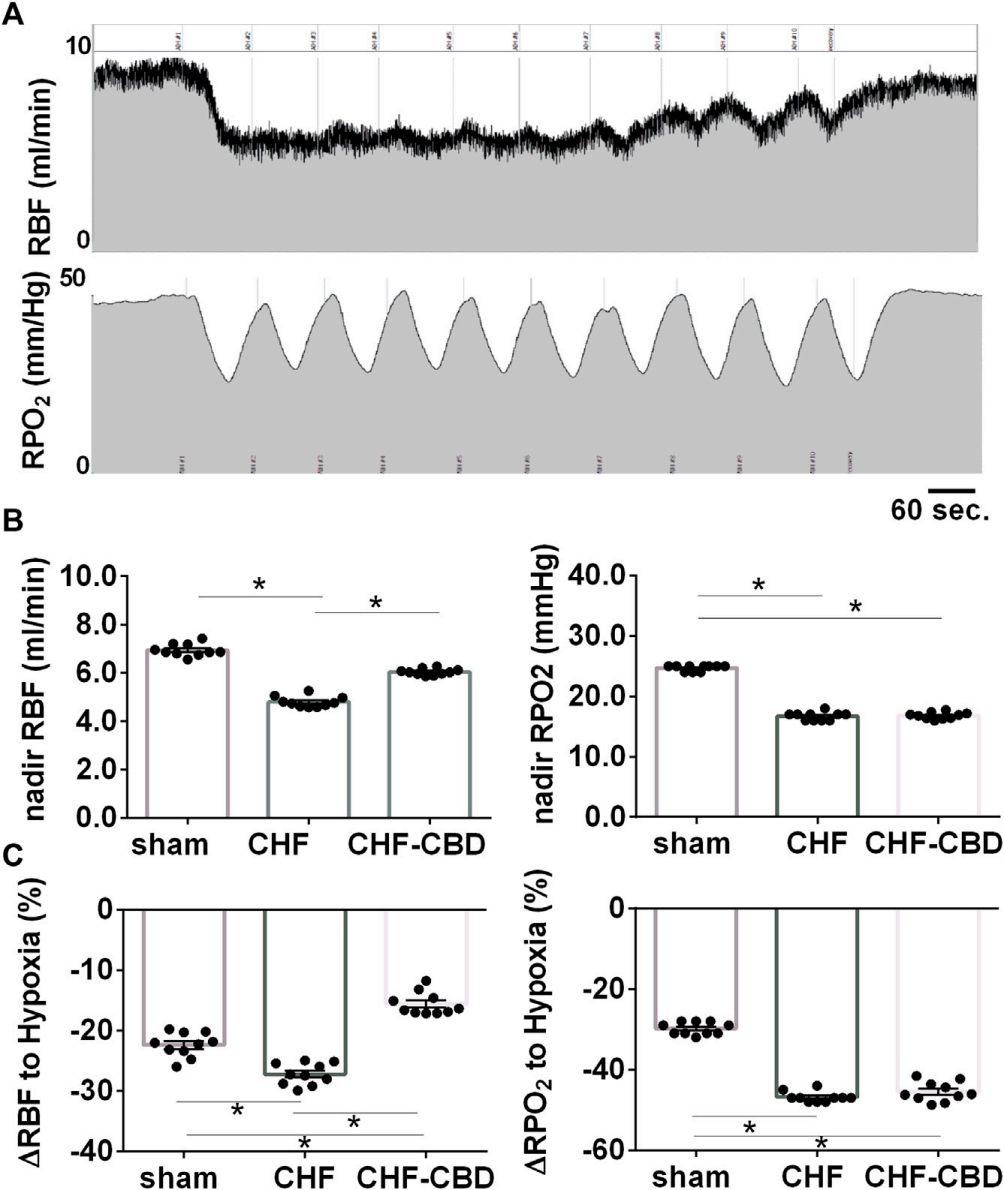
Effect of Intermittent Asphyxia (IA) on Control of Renal Blood Flow and Renal PO2 in CHF. Panel **(A)** illustrates representative tracings for RBF(top), and cortical RPO2 (bottom) responses to IA (10% FiO2, 3% FiCO2). Panel **(B)** contains summary data analyzing the mean nadir RBF and RPO2 during IA, and panel **(C)** contains summary data analyzing the mean change from baseline to nadir during IA. RBF and RPO2 were lower in CHF vs. sham and CBD attenuated the effect of CHF on RBF but not cortical RPO2. Similarly, the change in RBF and RPO2 were greater in CHF vs. sham, but CBD did not have a salutary effect on the change in RPO2. Data was analyzed using ANOVA with Sidak-Holm test for multiple comparisons. Results are expressed as mean ± SEM, *n* = 10 animals per group. *p < 0.05.

To determine if the CBC activation that occurs during central sleep apnea adversely affects control of RBF and RPO2, we exposed rats to a series of brief exposures to 10% O2, 3%CO2 (intermittent asphyxia, IA) while measuring RBF and RPO2. As show in Figure 5, nadir RBF and RPO2 during IA were significantly lower in CHF than sham (*p* < 0.05), and this effect was attenuated (*p* < 0.05) by CBD for RBF (RBF 7.0 ± 0.1 ml/min sham, 4.8 ± 0.1 ml/min CHF, 6.0 ± 0.1 ml/min CHF-CBD, F 257.6 DF 2,27) but not RPO2 (25 ± 0.2 mmHg sham, 17 ± 0.2 mmHg CHF, 17 ± 0.2 mmHg CHF-CBD, F 634 DF 2,27). Similarly, the mean change in RBF and RPO2 during IA was significantly greater (*p* < 0.05) in CHF vs. sham, and this effect was attenuated (*p* < 0.05) for RBF (−22 ± 0.6% sham, −27 ± 0.5% CHF, −15 ± 0.5% CHF-CBD, F 47.36 DF 2,27) but not RPO2 (−30 ± 0.5% sham, −47 ± 0.4% CHF, −45 ± 0.7% CHF-CBD, F 266.1 DF 2,27).

### Effect of carotid body denervation on renal cortical gene expression in chronic heart failure

**FIGURE 6:**
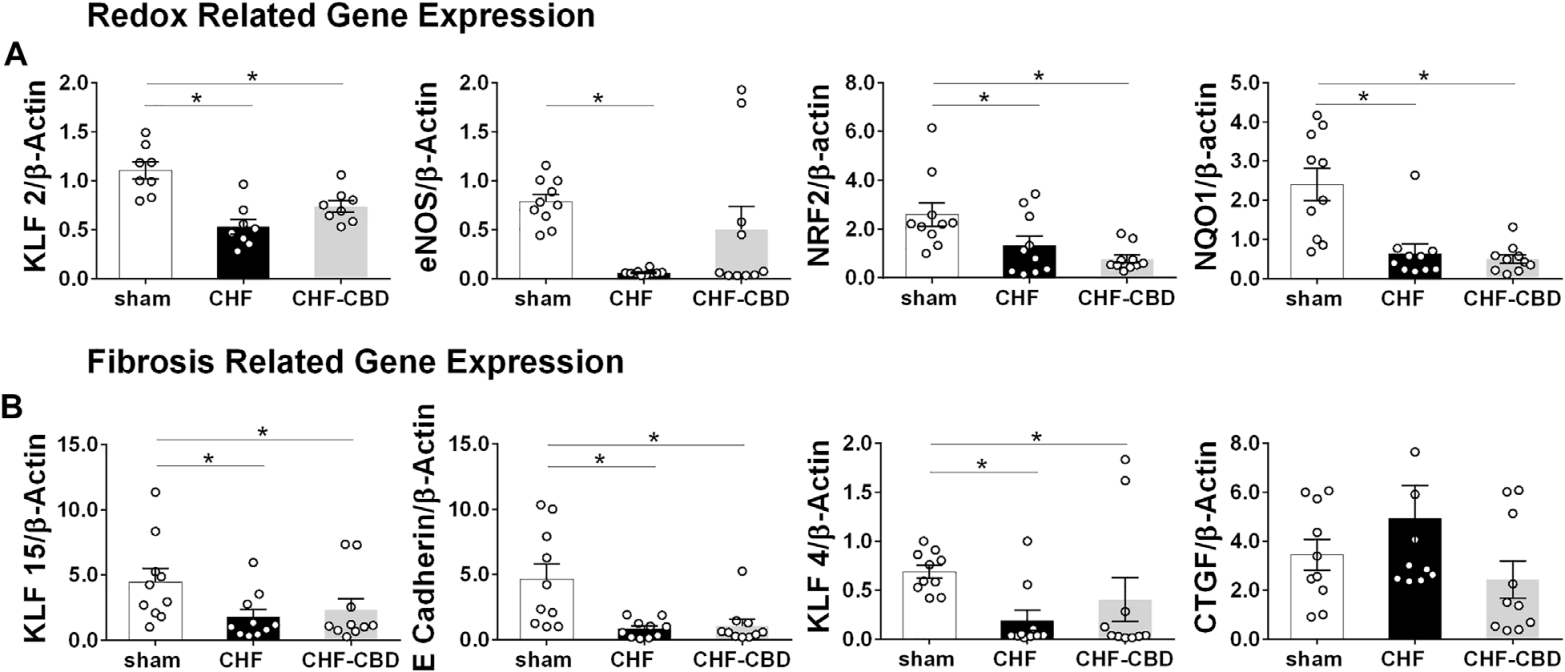
Effect of CHF and CBD on Redox and Fibrosis Related mRNA Expression in Renal Cortical Tissue. Panel **(A)** shows summary data from qRT-PCR assessing redox related mRNA expression in renal cortical tissue. KLF2, eNOS, NRF2, and NQO1 mRNA were significantly decreased in CHF compared to sham, however CBD did not restore expression of these genes. Panel **(B)** shows summary data from qRT-PCR assessing fibrosis related mRNA expression in renal cortical tissue. KLF15, E Cadherin, and KLF4 mRNA were significantly decreased in CHF compared to sham, and CBD did not restore expression of these genes. CTGF expression was not significantly different between groups. Results are expressed as mean ± SEM. n = 8–10 animals per group. *p < 0.05 via ANOVA or Kruskal Wallis with Sidak-Holm or Dunn’s multiple comparison tests, as appropriate. ANOVA: eNOS, KLF2. Kruskal Wallis: e Cadherin, KLF15, KLF4, NRF2, NQO1, CTGF.

Renal mRNA expression was assessed in cortical tissue using qRT-PCR. As shown in Figure 6A, redox-related genes KLF2 (1.11 ± 0.09 f.c. sham, 0.53 ± 0.08 f.c. CHF, 0.74 ± 0.06 f.c. CHF-CBD, F 14.88 2,21), eNOS (0.79 ± 0.07 f.c. sham, 0.06 ± 0.01 f.c. CHF, 0.50 ± 0.24 f.c. CHF-CBD, F 6.61 2,27), NRF2 (2.60 ± 0.48 f.c. sham, 1.14 ± 0.29 f.c. CHF, 0.78 ± 0.16 f.c. CHF-CBD, KWS 10.75), and NQO1 (2.40 ± 0.42 f.c. sham, 0.65 ± 0.23 f.c. CHF, 0.50 ± 0.11 f.c. CHF-CBD, KWS 15.36) were all significantly downregulated in CHF and CHF-CBD tissue vs. sham (*p* < 0.05). Expression of these genes in CHF-CBD was not significantly different from CHF (*p* > 0.05). As shown in Figure 6B, mRNA expression of fibrosis repressing genes KLF15 (4.46 ± 1.02 f.c. sham, 1.80 ± 0.57 f.c. CHF, 2.33 ± 0.85 f.c. CHF-CBD, KWS 6.37), E Cadherin (4.65 ± 1.12 f.c. sham, 0.84 ± 0.22 f.c. CHF, 1.09 ± 0.48 f.c. CHF-CBD, KWS 11.82), and KLF4 (0.69 ± 0.06 f.c. sham, 0.19 ± 0.10 f.c. CHF, 0.40 ± 0.22 f.c. CHF-CBD, KWS 8.82) were reduced in CHF and CHF-CBD relative to sham, however CHF and CHF-CBD were not significantly different from each other (*p* > 0.05). CTGF expression (3.44 ± 0.63 f.c. sham, 4.94 ± 1.35 f.c. CHF, 2.43 ± 0.75 f.c. CHF-CBD, KWS 4.25) was not significantly different between any groups.

## Discussion

Our results confirm a role for aberrant CBC function in the reduction in RBF and RPO2 observed in experimental CHF. We show that ablation of CBC early in the progression of CHF is sufficient to improve resting RBF and RPO2 and prevent further decrements in RBF during IA. Furthermore, we observed that CHF was associated with downregulation of shear stress sensitive transcription factor KLF2 and its downstream targets NRF2 and eNOS, and that gene expression of fibrosis-repressing KLF15, KLF4, and e-cadherin were reduced in CHF. These results lend further support to the hypothesis that enhanced CBC tonicity plays a significant role in the reduction in RBF in CHF and that this reduction in RBF is associated with reduced RPO2 and alterations in gene expression that promote oxidative stress and development of fibrosis.

### Chemoreflex control of renal blood flow in CHF

Clinical studies suggest that RBF is reduced in patients with CHF and that renal function (i.e., GFR) is an independent predictor of patient outcome and may be useful in stratifying patient’s prognosis (Cody et al., 1988; Ljungman et al., 1990; Hillege et al., 2000). Evidence from animal studies supports the notion that RBF is chronically reduced in CHF and shows that renal denervation can preserve RBF and inhibit activation of the renin angiotensin system (Clayton et al., 2011). The results of the current study confirm the role of CBC in mediating reductions in resting RBF/RVC observed in an animal model of ischemic reduced ejection fraction CHF. The relationship between RSNA and the control of RBF during chemoreceptor stimulation in healthy animals has been extensively characterized in the literature (Karim et al., 1987; Leonard et al., 2001; Evans et al., 2007). We have shown that the RSNA response to hypoxia is enhanced in CHF (Sun et al., 1999; Marcus et al., 2014; Marcus et al., 2018), and that the reduction in RBF in response to hypoxia is exaggerated in CHF (Marcus et al., 2015; Pügge et al., 2016). The finding that enhanced chemoreflex sensitivity contributes to tonic and hypoxia-evoked reductions in RBF is of potential clinical significance because patients with CHF frequently have comorbid sleep disordered breathing of both obstructive and central origin (Naughton et al., 1995; van de Borne et al., 1998; Giannoni et al., 2008). Our results suggest that the CBC can have a deleterious impact on chronic reductions in RBF with further superimposed periodic reductions in RBF associated with hypoxic apneic events and/or oscillatory breathing. This notion is supported by clinical evidence that preventing sleep disordered breathing in patients with CHF using adaptive servo-ventilation results in improvements in indices of renal function (Koyama et al., 2011; Owada et al., 2013; Yoshihisa et al., 2013).

Chronic activation of renal sympathetic nerves, RAAS activation, and renal hypoperfusion have been theorized to be important contributing factors in the development of the cardio-renal syndrome (Zannad and Rossignol, 2018; Costanzo, 2022; Patel et al., 2022). Our previous work and that of others has shown that global and renal sympathetic nerve activity are increased in CAL-induced CHF in rats and pacing-induced CHF in rabbits (Del Rio et al., 2013; Marcus et al., 2014; Wang et al., 2014; Sharma et al., 2016; Marcus et al., 2018). In the pacing-induced CHF model we have shown that RSNA is decreased by 42% in CHF-CBD animals relative to CHF(8). In addition, in this same model we have shown that RBF is reduced in CHF and that RBF is 31% higher in CHF-CBD animals (Marcus et al., 2015). The current study did not specifically address the role of a reduction in RBF on RAAS activation in CHF; however, previous studies have shown that CHF is associated with decreased RBF (Clayton et al., 2011), increased ANG II type 1 receptor (AT1R) expression and decreased ANG II Type 2 receptor expression in the kidney (Clayton et al., 2011; Peng et al., 2020) and that reducing sympathetic stimulation of the kidney by renal denervation prevents these changes (Clayton et al., 2011). Thus our current and previous (Marcus et al., 2015; Pügge et al., 2016) findings suggest that CBC activation in CHF may contribute to development of cardio-renal syndrome by increasing RSNA and decreasing RBF.

### Renal blood flow and renal PO2 in chronic heart failure

Renal tissue PO2 has been extensively studied as a potential contributor to renal injury and dysfunction in the context of chronic kidney disease and hypertension (Honda et al., 2019). Renal tissue hypoxia can theoretically result from decreased oxygen (O2) delivery (e.g., hypoperfusion) or as a result of increased O2 consumption, as with inflammation or increased sodium reabsorption (Honda et al., 2019). Nitric oxide (NO) plays a key role in regulation of tissue O2 supply and metabolism in the kidney and the effects of NO are opposed by Angiotensin II (Rajapakse et al., 2008). Decreased renal NO bioavailability and increased renal Angiotensin II, as has been shown in CHF kidneys (Abassi et al., 1997), would have a net effect of increasing renal O2 consumption and pre-disposing to renal hypoxia. The presence of renal hypoperfusion, RAAS activation, decreased NO bioavailability (secondary to oxidative stress), inflammation, and increased sodium retention in CHF increase the likelihood of chronic renal tissue hypoxia. Our study shows for the first time that RPO2 is reduced in CHF and that this reduction is associated with chemoreflex function, as it was attenuated by bilateral CBD.

### Central sleep apnea, chemoreflex function, and renal function in chronic heart failure

Many CHF patients experience obstructive and central apneas during sleep (Naughton et al., 1995; Giannoni et al., 2008; Javaheri et al., 2017), and otherwise healthy patients with sleep apnea have enhanced CBC-mediated sympathetic activation in response to hypoxia (Narkiewicz et al., 1999; Javaheri et al., 2017). In addition to CHF-related factors that enhance CBC sensitivity, the intermittent asphyxia (IA) associated with sleep apnea exacerbates aberrant CBC function in CHF. Numerous studies show that the IA associated with sleep apnea results in increases in afferent CBC activity (Peng et al., 2003; Del Rio et al., 2014) that contributes to elevated SNA during normoxia and during hypoxic stimulation (Marcus et al., 2010; Javaheri et al., 2017) and that this has downstream effects on RBF (Marcus et al., 2015; Pügge et al., 2016). These factors contribute to decrements in renal function as sleep apnea is independently associated with decreased renal function in aging populations and is prevalent in patients with end-stage renal disease (Canales et al., 2008; Lin et al., 2020). Severity of sleep apnea is highly correlated with markers of renal damage and is associated with enhanced chemoreflex sensitivity (Narkiewicz et al., 1999; Canales et al., 2008; Lin et al., 2020). Episodes of sleep apnea are expected to further exacerbate increases in RSNA and reductions in RBF and RPO2. In addition to hypoperfusion, the hypoxemia associated with IA would be expected to present an additional insult and further decrease RPO2. This study shows for the first time that intermittent asphyxia in CHF has an additive effect as it pertains to reductions in RBF and RPO2. In our study we observed that IA further exacerbated the effects of enhanced CBC sensitivity in CHF on RBF confirming the deleterious physiological effects of this additional insult in CHF.

In contrast to RBF, we did not observe an effect of CBD to improve RPO2 during IA. We believe this is in part a function of using bilateral CBD for our intervention. While we did not measure ventilation during our measurements of RBF and RPO2, bilateral CBD likely resulted in an inadequate ventilatory response to hypoxia that counteracted the potential beneficial effect of improvements in RBF. This theory would be consistent with our previous observations in this model (Del Rio et al., 2013). For this reason, interventions that normalize CBC sensitivity without eliminating it may result in beneficial effects that are not observed with bilateral CBD. In support of this notion, Niewinski et al. (2017) showed that unilateral carotid body resection in patients with systolic heart failure reduced the ventilatory response to hypoxia (without eliminating it) while also eliciting a significant reduction in sympathetic nerve activity. Also of particular relevance to our findings, bilateral carotid body resection in patients with systolic heart failure resulted in a trend towards worsening oxygen saturation at night that was not observed in patients undergoing unilateral carotid body resection (Niewinski et al., 2017).

### Molecular mechanisms contributing to renal dysfunction in chronic heart failure

Because of our particular interest in changes in sympathetically mediated decreases in RBF we interrogated gene expression of transcription factors related to blood flow. Also, because CHF is associated with a pro-oxidative state and based on previous studies in which CHF was associated with downregulation of antioxidant genes (Ding et al., 2009; Lu et al., 2022), we sought to determine if renal antioxidant gene expression was altered in our model of CHF and if CBD would have any salutary effect. In particular we were interested in expression of the shear stress-sensitive transcription factor Kruppel-like Factor 2 (KLF2), as KLF2 controls expression of mediators of antioxidant defense, nitric oxide production, and tissue fibrosis (Dekker et al., 2005; Boon et al., 2007; Fledderus et al., 2008) and has previously been shown to be downregulated in animal models of CHF (Haack et al., 2014; Del Rio et al., 2017; Marcus et al., 2018). As shown in Figure 5A, we observed that the gene expression of renal cortical KLF2 and the master antioxidant transcription factor NRF2 (the downstream target of KLF2) were significantly downregulated in CHF. To determine if NRF2 downregulation resulted in decreased gene expression of antioxidant enzymes under its control we measured mRNA expression of NQO1, which was also significantly decreased in CHF. KLF2 also controls expression of endothelial nitric oxide synthase (eNOS), and because NOS-derived NO plays a critical role in redox balance and oxygen consumption, we measured eNOS mRNA expression in renal cortex. As expected, we observed that renal cortical eNOS mRNA expression was significantly lower in tissue from CHF animals. This is particularly relevant as loss of renal cortical NO derived from eNOS could contribute to decrements in RPO2 observed via increased oxygen consumption (Emans et al., 2018).

Despite improvements in RBF that occurred with CBD, we did not observe significant increases in expression of KLF2, NRF2, NQO1, or eNOS mRNA in CHF CBD tissue, suggesting that either the improvement in RBF and RPO2 was not sufficient to reverse changes in gene expression or that additional factors not affected by CBD impinge on these pathways. Indeed there is evidence that NRF2 is regulated by circulating microRNA contained within extracellular vesicles released from cardiac tissue in CHF, and that these extracellular vesicles may mediate inter-organ communication w/respect to NRF2 activity/expression (Tian et al., 2021). CBD did not improve cardiac function within the time frame of our study thus it is plausible that renal NRF2 gene expression could be affected by cardiac dysfunction via extracellular vesicle encapsulated microRNA. While this question is outside of the scope of the current investigation, the hypothesis deserves further study.

Tissue fibrosis has been theorized to be a common pathway in cardio-renal syndromes (Zannad and Rossignol, 2018) and so we examined gene expression of fibrosis related pathways in renal cortical tissue. In particular we focused on pathways related to tissue hypoxia because of its relevance to our physiological measurements and on RAAS activation because it is a hallmark of CHF that is related to renal nerve activity and blood flow. To this end we measured gene expression of Kruppel-like factor 15 (KLF15), Kruppel-like factor 4 (KLF4), E-Cadherin, and connective tissue growth factor (CTGF). KLF15, KLF4, and E-Cadherin, all of which have fibrosis suppressing activity, have been shown to be downregulated by Angiotensin II (ANG II) and hypoxia (Esteban et al., 2006; Lu et al., 2019; Yang et al., 2020). Therefore high ANG II levels expected in CHF and the decreased RPO2 we observed in our studies could promote renal fibrosis in part by suppression of KLF15, KLF4, and E--Cadherin. As shown in Figure 5B, gene expression of all three of these targets was significantly lower in CHF tissue relative to sham, however there were no significant differences between CHF and CHF-CBD groups for any of these targets. CTGF which is pro-fibrotic is induced by hypoxia and ANG II (Higgins et al., 2004; Rupérez et al., 2003) and suppressed by KLF15 (Gu et al., 2017) was not significantly different between any groups.

### Limitations

The CAL model of CHF is an accepted and widely used model of dilated cardiomyopathy of ischemic origin (Houser et al., 2012). Chronic heart failure is a condition that may be precipitated by a wide variety of pathophysiological insults including (but not limited to) hypertension, abnormal valvular function, viral myocarditis, cardiac toxicity due to alcohol or drug use, congenital heart defects, pulmonary dysfunction, obesity, diabetes, and/or sleep apnea. Our model therefore does not recapitulate all aspects of CHF of various origins and therefore the inference that can be drawn from our findings is limited to the scope of CHF of ischemic origin.

We employed CBD at an early time point in the experimental protocol. This allows us to make inferences about the influence of CBC activity on RBF and RPO2 in CHF, however it does not allow us to make inferences about the potential efficacy of this intervention if it were applied at a later time point. It should be noted though that previous studies from our laboratory have shown that CBD performed in the same animal model (ischemic) at later time points in the development of CHF has beneficial effects on autonomic function and survival (Del Rio et al., 2013), suggesting that CBD performed at later time points could have beneficial effects on RBF and RPO2. Control of renal blood flow is an integrated response that is composed of autocrine and paracrine influences on vascular function as well as the influence of sympathetic nerve activity. Similarly tissue PO2 is a function of both oxygen delivery and oxygen consumption. In this study we only measured the integrated responses of RBF and RPO2 and therefore cannot draw inferences about relative changes in nerve activity, vascular function, or oxygen consumption. With that said, previous studies in pacing-induced CHF rabbits have shown that CBD reduces renal nerve activity (Marcus et al., 2014), and that denervation of renal nerves results in improvements in renal blood flow (Clayton et al., 2011; Marcus et al., 2015), lending support to the notion that our results are at least in part a function of CBC mediated increases in renal nerve activity and attendant reductions in RBF and that the salutary effect of CBD is a function of reductions in RSNA. It is important to note when evaluating relationships between the experimental variables that some variables were measured under anesthesia and some were not (i.e., GFR).

We did not measure arterial PO2, SaO2, or hematocrit during these experiments and therefore we cannot make inferences or statements about changes in oxygen carrying capacity of the blood. Because of this, we cannot rule out the possibility that increases in arterial oxygen content influenced the improvements in RPO2 we observed (as opposed to the improvement in RBF alone). With that said, we feel that the observation of an improvement of RPO2 in CHF-CBD is the most important finding, regardless of whether or not it was a result of improved oxygen carrying capacity, improved RBF, or a combination of the two.

Finally, our results do not allow for us to account for the mRNA half-life of the different targets measured in our study. Therefore it is possible that the changes in gene expression observed could have been the result of CHF per se, IA per se, or CHF combined with IA. Also, we did not observe any differences in CTGF expression in our renal cortical tissue samples, however It is possible that changes in CTGF expression were washed out due to a mix of cell types in our tissue samples used for analysis. There is some evidence of differential regulation of CTGF expression in response to a given stimulus in different kidney cell types (Cicha and Goppelt-Struebe, 2009; Komorowsky et al., 2009), and therefore it is plausible that our technique lacked the required resolution to detect changes in CTGF expression.

### Clinical implications

Conventional CHF therapy is of limited utility in patients with CRS, and renal dysfunction is a prominent independent risk factor for all-cause mortality in CHF patients (Hillege et al., 2000; Zannad and Rossignol, 2018; Costanzo, 2022). Thus additional therapeutic approaches which attenuate renal dysfunction and prevent progression could have a substantial impact on quality of life and prognosis in this population. This study provides new insight into the mechanisms that affect renal function in CHF and the translational impact of CBD on these parameters. Modulation of carotid body activity has been an area of significant interest as a potential therapeutic approach for sympathetically mediated diseases in general (Paton et al., 2013) and CHF in particular (Niewiński et al., 2013; Niewinski et al., 2014; Niewinski et al., 2017), and our study identifies the potential utility of this approach in treatment of patients with CRS. As a result, therapeutic management of CHF patients may be adapted to incorporate strategies that reduce afferent CBC activity.

## Conclusion

Our results indicate that enhanced tonic CBC activity and sensitivity to IA contributes to reductions in RBF and RPO2 in CHF which are coincident with changes in renal gene expression that promote oxidative stress and fibrosis. Elimination of CBC input by CBD attenuated reductions in RBF and RPO2 which could contribute to development of CRSII in CHF. These findings suggest that enhanced CBC tonicity plays a role in altered renal hemodynamics and tissue hypoxia in CHF and that therapeutic modalities that normalize CBC function may improve renal hemodynamics and RPO2 and decrease related tissue injury.

## Data Availability

The raw data supporting the conclusions of this article will be made available by the authors, without undue reservation.
